# Cognitive Performance in Men and Women Infected with HIV-1

**DOI:** 10.1155/2013/382126

**Published:** 2012-12-26

**Authors:** José María Faílde Garrido, María Lameiras Fernández, Marika Foltz, Yolanda Rodríguez Castro, María Victoria Carrera Fernández

**Affiliations:** Departamento de Análisis e Intervención Psicosocioeducativa, Facultad de Ciencias de la Educación, Universidad de Vigo, Campus Universitario As Lagoas, Avenida Castelao s/n, 32004 Ourense, Spain

## Abstract

*Introduction*. Very few studies have examined the neuropsychological performance of HIV-positive women, and even fewer have attempted a comparison of cognitive functioning by gender. The aim of this study was to describe the nature of the neuropsychological performance of HIV seropositive patients by gender. *Methods*. A clinical sample made up of 151 subjects was recruited to participate in this study. All of the subjects underwent the same assessment process, consisting of a neuropsychological evaluation and an interview to gather sociodemographic, toxicological, and clinical data. *Results and Discussion*. Despite the fact that men obtained higher scores in visual memory, attention/psychomotor speed, and abstract reasoning/verbal intelligence, these differences were not statistically significant. In contrast, significant differences were found depending on subjects' serological status. Seropositive participants' neuropsychological performance was significantly lower than that of the seronegative participants in all of the areas assessed as follows: (1) visual memory; (2) attention/psychomotor speed; (3) abstract reasoning/verbal intelligence; (4) verbal memory for texts; (5) verbal memory for digits and words. *Conclusions*. The results from this study reveal no significant gender differences in the cognitive performance of patients infected with HIV-1.

## 1. Introduction

From a scientific standpoint, women have traditionally been underrepresented in biomedical and psychological research [1]. This has also been the case in studies done on the neurological consequences of HIV-1, in which the majority of research to date has been carried out with all-male samples [2, 3]. Although this tendency has begun to change in recent years, there is still only a limited number of studies which examine neuropsychological aspects of seropositive women, or which compare the cognitive functioning of seropositive patients by gender [4, 5]. As a consequence, we still know very little about the neuropsychological consequences of the HIV-1 virus for seropositive women. 

The scarce information which is available does not shed much light on the question; contradictory results have been found regarding the existence of differences in neuropsychological functioning between seropositive and seronegative women [6, 7]. For instance, Mason et al. [8] found differences between HIV-infected and noninfected women in the areas of psychomotor speed, verbal memory, and attention. In contrast, other researchers did not find such differences [9, 10]. Durvasula et al. [6] found differences between seropositive women (with and without AIDS) and seronegative women in the functions of psychomotor speed, verbal memory, and motor speed. However, in their comparisons of AIDS patients and seropositive asymptomatic subjects, the only difference found was in psychomotor speed. In another study carried out by Richarson et al. [11], no significant differences were found in terms of neuropsychological performance between seronegative and seropositive women when the latter were being administered antiretroviral therapy, while such differences were found when the HIV-infected subjects were not being medicated. 

Numerous studies have found differences between seronegative women and men, in hormone levels [12], chemistry and brain structure, body composition, behaviour, and drug addiction [13]. Other studies have found that seropositive women, as compared to their male counterparts, more often present a history of substance abuse [14], tend to have lower incomes [15], present a greater number of psychiatric problems, show a faster development of the disease as well as higher mortality rates [16], and tend to have attained lower levels of formal education [17]. All of these aspects could, theoretically, explain the differences found in the neuropsychological performance of HIV-positive men and women [18].

Given this situation, it would be reasonable to expect greater neuropsychological vulnerability in seropositive women than in seropositive men [19], as well as different characteristics in terms of their neuropsychological functioning. However, the few studies which have undertaken a comparison of HIV-positive women and men have not found important differences in their neuropsychological performance [20, 21].

The incidence of HIV infection among women continues to grow [22]. For this reason, neuropsychological research should include both women and men in its samples, and furthermore, it should analyse the data by gender [23]. The objective of the present study is to describe the nature of the neuropsychological performance of patients infected with HIV-1 and to compare the resulting data by gender. 

## 2. Methods

A total of 151 heterosexual subjects participated in this study. The sample was comprised of both women and men, 90 of whom were seropositive and 61 seronegative. All of them gave their informed consent before the start of the research. The sample was divided into four groups: (1) seropositive men  (*n* = 57); (2) seropositive women (*n* = 33); (3) seronegative men (*n* = 31); (4) seronegative women (*n* = 30). 

The seropositive sample was recruited from among the patients who received regular medical attention in the centre for infectious diseases at the Cristal-Piñor Hospital Complex in Ourense (Spain). In order to be considered apt for inclusion in this study, the patients were required to: be infected with the HIV-1 virus; not be coinfected with the HIV-2 virus; not have been hospitalised in the previous 30 days; not have consumed drugs in the previous three months (although participating in maintenance programmes using methadone was considered acceptable); not have shown signs of severe, chronic alcohol abuse in the previous six months; not be infected with other disorders of the CNS caused by systemic disorders or opportunistic pathogens or neoplasias; not display disorders of the peripheral nervous system or of the muscles which could affect their performance on certain neuropsychological tests; not have a history of severe neurological or psychiatric disorders, or ones which would require psychopharmacological treatment, or which might alter their level of consciousness or behaviour in any way. 

In order to recruit subjects for the control group (seronegative), participants were selected from the same social or family milieu as those in the seropositive sample, as well as individuals who were considered to be at risk of being infected with the HIV-1 virus. Those with a history of drug dependency were excluded from the sample. The control group was matched as far as possible to the seropositive group in terms of age, gender, and years of education. 

All of the participants who were finally selected to take part in the study were told about the purpose of our research and given an appointment at which to undergo the neuropsychological assessment. In order to minimise time and costs, the appointments were set up to coincide with their regular medical check-ups in the case of seropositive participants. In this way, with patients' consent and their physicians' collaboration, we were able to access information regarding their current levels of CD4, viral load, and other clinical and biological parameters. 

All of the participants underwent the same assessment process, which consisted of: a semi-structured interview with questions pertaining to relevant socio-demographic, toxicological, biological, and clinical data; neuropsychological assessment via a non-standardised battery designed *ad hoc* for this study. 

The neuropsychological battery was made up of a compendium of tests, all of which have a long tradition and were chosen for their validity and because they have been shown to be sensitive to the assessment of neuropsychological functioning in HIV-infected patients in others studies. The tests covered a wide range of cognitive functions, which were regrouped based on the results obtained from carrying out a factor analysis (Varimax with Kaiser method), thus identifying five factors which explained 73.34% of the variance, as has been reported in a previously published study [24]. These factors were named based on the characteristics of the tests which they contained: (1) visual memory: The Spanish version of the Benton Visual Retention Test-Form C, Administration A; correct and errors [25],  and the Spanish version of the Rey-Osterrith Complex Figure Test—Copy and Delay [26]; (2) attention/psychomotor speed: The Trail Making Test [27], The Arithmetic Subscale of the Spanish version of WAIS-R [28], and the Spanish version of the Toulouse-Pieron Test [29]; (3) abstract reasoning/verbal intelligence: The Vocabulary, Comprehension, and Similarities subtests of the Spanish version of WAIS-R [28]; (4) verbal memory for texts: Babcock Story Recall Test—Immediate and Delayed; Version translated into Spanish [30]; (5) verbal memory for digits and words: the Spanish version of the Rey Auditory Verbal Learning Test (Correct and Errors) [31], and the Digit Span subtest of the Spanish version of WAIS-R [28]. 

## 3. Results

As regards the similarity of male and female participants in the seropositive and seronegative groups, no significant differences were found between men and women in socio-demographics or clinical aspects in the seropositive sample or in the seronegative sample, as can be seen in Table 1. 

Before undertaking the evaluation of subjets' neuropsychological performance, it was necessary to transform the direct scores into standardised scores for each of the tests included in the five functions studied. In this way each test carried the same weight in relation to the global score for the corresponding factor. 

In order to examine the effect of gender and seropositivity on neuropsychological performance, three levels of analysis were carried out: (1) an intergender comparison for seronegativity: seronegative men as compared to seronegative women; (2) an intergender comparison for seropositivity: seropositive men versus seropositive women; (3) intragender comparisons: seropositive women versus seronegative women; and seropositive men versus seronegative men. 

**Figure 1:**
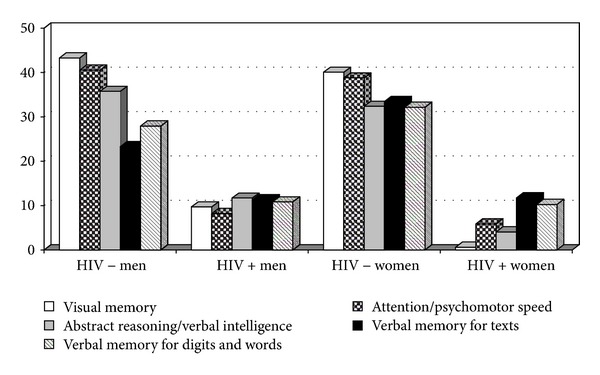
Neuropsychological performance for seropositive and seronegative samples.

The results from the first group of data analysed, involving the differences between male and female HIV-negative participants, can be seen in Figure 1. Despite the visible differences in neuropsychological performance between seronegative women and men in the five factors analysed, no significant differences were found for any of them (visual memory: *T* = 0.56, *P* > 0.956; attention/psychomotor speed: *T* = −0.19, *P* > 0.849; abstract reasoning/verbal intelligence: *T* = 0.74, *P* > 0.941; verbal memory for texts: *T* = −1.96, *P* > 0.610; verbal memory for digits and words: *T* = −0.54, *P* > 0.595). 

The second group of data involved a comparison of seropositive men and women in terms of their neuropsychological performance (see Figure 1). The most salient result is the low scores obtained by all of the seropositive participants in all five functions studied, while no significant differences were found between the two genders. If we look more closely at the results, we find that men presented higher levels of performance in visual memory, attention/psychomotor speed, and abstract reasoning/verbal intelligence, whereas women scored higher than men in verbal memory for texts. However, we must bear in mind that the differences were not statistically significant in either case (visual memory: *T* = 1.33, *P* > 0.187; attention/psychomotor speed: *T* = 0.33, *P* > 0.743; abstract reasoning/verbal intelligence: *T* = 1.35, *P* > 0.179; verbal memory for texts: *T* = −0.12, *P* > 0.907; verbal memory for digits and words: *T* = 0.13, *P* > 0.895). 

The third and final group of data involved an intragender analysis. With the aim of separating the influence of gender and seropositivity, we compared seropositive women with seronegative women, and seropositive men with seronegative men. As expected, both HIV-positive women and men obtained significantly lower scores on the neuropsychological tests than their HIV-negative counterparts (see Figure 1). 

For the male participants, significant differences were found in all five functions depending on their serological status: visual memory (*T* = −4.50, *P* < .000); attention/psychomotor speed (*T* = −3.99, *P* < .000); abstract reasoning/verbal intelligence (*T* = −3.79, *P* < .000); verbal memory for texts (*T* = −2.80, *P* < .006); verbal memory for digits and words (*T* = −2.89, *P* < .005). Similarly, significant differences were found between seropositive and seronegative female participants in all five factors studied: visual memory  (*T* = −3.27, *P* < .002); attention/psychomotor speed (*T* = −3.14, *P* < .003); abstract reasoning/verbal intelligence (*T* = −3.06, *P* < .004); verbal memory for texts (*T* = −3.10, *P* < .004); verbal memory for digits and words (*T* = −2.78, *P* < .008).

## 4. Discussion

From an intergender perspective, the data indicate an absence of statistically significant gender differences in all of the factors studied, both for the group of seronegative patients and for the seropositive patients. These results are in line with findings from some previous studies [9, 10], while they are at odds with findings reported by Durvasula et al. [6] and Lopez et al. [7]. Along similar lines, other studies indicate that, although there are no gender differences in general intelligence, differences are found on some tasks measuring cognitive abilities [32]. In general, women score higher on tasks that require rapid access to and use of phonological and semantic information in long-term memory, production and comprehension of complex prose, fine motor skills, and perceptual speed, while men tend to obtain higher scores on tasks that require transformations in visual-spatial working memory, motor skills involved in aiming, spatiotemporal responding, and fluid reasoning, most notably in abstract mathematical and scientific tasks [32, 33].

As explained earlier, the intergender comparison was accompanied by another level of analysis in which women and men were compared separately (intragender), taking into account their serological status to determine whether any differences could be found; we considered this advisable given the recommendations stemming from research comparing neuropsychological performance in seropositive patients by gender [4, 6, 23]. For this reason, we undertook to analyse the neuropsychological performance of HIV-positive and HIV-negative men and women and to compare them with their counterparts with the same serological status. This allowed us to separate the effect of gender from that of seropositivity. We must bear in mind that being infected with HIV-1 often implies simultaneous life circumstances (e.g., a history of substance abuse, a low level of formal education, a low income, etc.) which may collectively contribute to or help explain the neuropyschological deterioration found in seropositive subjects in this type of study [2].

Our analysis of the specific characteristics associated with the neuropsychological performance of HIV-positive and HIV-negative subjects from an intragender perspective reveals significant differences between the groups, both for women and men. These results are in line with those cited in studies which have found a link between seropositivity and low levels of neuropsychological functioning, both with female samples [6, 8] and male samples [2, 33, 34]. However, our findings do not concur with those found in some other studies in which no connection was found between seropositivity and neuropsychological performance [9, 11, 35, 36], although in these four studies cited, the samples were made up exclusively of women. 

Despite the lack of significant differences found between genders in both the seronegative and seropositive samples, there appears to be a greater gap between genders in the group of subjects infected with the HIV-1 virus. This leads us to consider the possibility that seropositivity—along with all of the life circumstances that accompany it—may be a factor which negatively affects cognitive functioning. Another interesting observation is that the overall neuropsychological performance patterns observed appear to differ by gender. This may offer at least partial confirmation of what Satz et al. [19] claim regarding a greater vulnerability to neuropsychological disorders in seropositive women than in seropositive men. Nonetheless, there is clearly a need for further research on the topic in order to confirm this observation. 

Finally, it should be pointed out that our study presents certain limitations, mainly due to the sample size used for the control groups. If the control groups had been larger, it would have allowed us to use multivariate statistical techniques. By doing so, we could have analysed the possible effects or interactions of other variables which were not considered in the present study. Clearly, more research is needed on this subject—using larger samples—to clarify the relationship, if any, between gender and neuropsychological performance in patients infected with HIV-1.

## 5. Conclusions

The present research is one of the few international studies which have examined neuropsychological performance from a gender perspective. Based on the results obtained, we can affirm that no significant differences were found in neuropyschological performance when comparing subjects by gender, but such differences were clear when analysing the data in terms of serological status. While gender did not prove to be conclusively linked to neuropsychological performance in this study, we would like to suggest that future research continue to examine possible differing patterns of cognitive functioning between men and women. 

## Figures and Tables

**Table 1:** Sociodemographic, biological, and clinical characteristics of seropositive and seronegative subjects by gender.

		HIV+					HIV−				
Variable		Men (*n* = 57)		Women (*n* = 33)		*F*/*χ* ^2^ value	Men (*n* = 30)		Women (*n* = 31)		*F*/*χ* ^2^ value
		Mean	(SD)	Mean	(SD)	*F*	Mean	(SD)	Mean	(SD)	*F*
Age		34.17	(5.33)	33.42	(5.06)	.017	31.56	(6.57)	35.04	(5.08)	.545
Years of education		9.32	(2.42)	10.45	(3.18)	.260	10.02	(2.38)	9.66	(2.49)	.238
CD4 cells per m^3^		286.68	(261.59)	367.43	(220.67)	2.131	—	—	—	—	—

Variable		No.	(%)	No.	(%)	*χ* ^2^	No.	(%)	No.	(%)	*χ* ^2^

Manual dominance	Right	57	(100.00)	31	(93.94)	.048	16	(88.88)	14	(87.50)	.003
	Left	0	(0.00)	2	(6.06)		2	(11.12)	2	(12.50)	
Drug	Never used drugs	12	(21.10)	10	(30.03)	.063	18	(100.00)	16	(100.00)	.000
Abuse	Abstinent IVDU*	25	(43.80)	8	(24.24)		—	—	—	—	
Status	MMP*	20	(35.10)	15	(45.45)		—	—	—	—	
Viral load	Not detectable	14	(24.60)	11	(33.30)	1.214	—	—	—	—	—
	Low (<10,000)	19	(33.30)	11	(33.33)		—	—	—	—	
	Medium (from 10,000–30,000)	7	(12.30)	3	(9.09)		—	—	—	—	
	High (>30,000)	17	(29.80)	8	(24.25)		—	—	—	—	
Antiretroviral therapy	Receiving	55	(96,49)	32	(96,96)	.000	—	—	—	—	—
	Not receiving	2	(3.51)	1	(3,03)		—	—	—	—	
Phase of infection	Phase A	16	(28.10)	14	(42.42)	1.281	—	—	—	—	—
	Phase B	13	(22.80)	6	(18.18)						
	Phase C	28	(49.10)	13	(39.40)						

^∗^ *P* < .05, ^∗∗^ *P* < .001.

*Note: IVDU: Intravenous Drug User; MMP: Methadone Maintenance Programme.
